# Integrative Taxonomy Clarifies the Taxonomic Status of the Morphologically Intermediate Form Between *Tropidothorax cruciger* and *T. sinensis* (Hemiptera: Lygaeidae)

**DOI:** 10.3390/insects17010037

**Published:** 2025-12-26

**Authors:** Chenguang Zheng, Xiuxiu Zhu, Yaning Zhang, Ying Wang, Wenjun Bu

**Affiliations:** 1 School of Public Health, North China University of Science and Technology, Tangshan 063210, China; cgzheng@ncst.edu.cn; 2 Institute of Entomology, College of Life Sciences, Nankai University, 94 Weijin Road, Tianjin 300071, China; 3 Department of Neuroscience, Faculty of Life Sciences, University College London, London WC1H 0AP, UK

**Keywords:** species delimitation, DNA barcoding, genome-wide SNPs, intraspecific variation

## Abstract

(1) Background: The identification of *Tropidothorax cruciger* and *T. sinensis* is often complicated by the presence of the “intermediate form”. Due to the lack of molecular data, the taxonomic status of the “intermediate form” and the species boundaries between *T. cruciger* and *T. sinensis* remain uncertain; (2) Methods: In this study, we integrated morphological, molecular, and ecological data to delimit species boundaries of these two species using multiple species delimitation approaches; (3) Results: Most species delimitation analyses based on the cytochrome c oxidase subunit I (COI) fragment suggested that *T. cruciger* and the “intermediate form” comprised a single species, with *T. sinensis* representing a separate species. This delimitation result was also supported by the analyses of BFD* and genetic clustering based on genome-wide SNPs. Under this species delimitation scenario, a clear-cut barcode gap was discovered between the interspecific and intraspecific genetic distances. In addition, environmental-related analyses showed highly similar ecological requirements of *T. cruciger* and the “intermediate form”, supporting their recognition as a single species; (4) Conclusions: This study clarifies the taxonomic status of the “intermediate form” and the species boundaries between *T. cruciger* and *T. sinensis*, which is essential for further studies of ecology and evolution of these species.

## Simple Summary

The presence of the “intermediate form” between *Tropidothorax cruciger* and *T. sinensis* has complicated the identification of these two species. In this study, we performed an integrative taxonomy combining morphological features with molecular and ecological data. The taxonomic status of the “intermediate form” and the species boundaries between *T. cruciger* and *T. sinensis* were clarified. The “intermediate form” should be recognized as a morphological variant of *T. cruciger*. A clear barcode gap was found between interspecific and intraspecific genetic distances of *T. cruciger* and *T. sinensis*. Our study provides a robust framework for the accurate delimitation of these two species.

## 1. Introduction

Species are the fundamental biological units, and the accurate delimitation of species is the primary step in the study of biodiversity, systematics and evolution [1,2,3]. Morphology-based taxonomy classifies species based on external morphological features or reproductive structures, which is the cornerstone of taxonomic science [4,5]. However, morphology-based taxonomy often faces challenges in accurately delimiting species boundaries for closely related species with similar appearances [6,7,8,9]. Moreover, in groups with high intraspecific variation, relying solely on morphological traits may lead to the over-splitting of species [10,11]. In such cases, advanced molecular techniques and modern tools can be very helpful. By analyzing DNA sequence variation, molecular-based species delimitation approaches can provide preliminary taxonomic hypotheses and compensate for the limitations of traditional morphology-based taxonomy [12,13,14,15]. In recent years, the employment of molecular-based delimitation methods has been increasingly recognized as an essential tool in taxonomy and systematics and has played a significant role in clarifying species boundaries, especially in groups characterized by high levels of intraspecific variation [16,17,18,19,20,21,22].

The most commonly used molecular markers include the mitochondrial genes, nuclear gene fragments, and genome-wide single-nucleotide polymorphisms (SNPs) [23,24,25]. Mitochondrial genes, specifically the COI gene fragment used as the standard animal DNA barcode, are known to be powerful molecular markers for delimiting species boundaries. The single-locus barcoding approach serves as a valuable tool for the preliminary delimitation of species boundaries in poorly known taxa, and as a key supplement to traditional morphology-based taxonomy in morphologically conserved taxa [26,27,28]. Recently, next-generation sequencing technologies have enabled the generation of vast amounts of genetic data—such as thousands of genome-wide SNPs—greatly enhancing the study of insect systematics [29]. Due to the abundance of genetic information, genome-wide SNPs have been widely employed to infer phylogenetic relationships and to delimit species among closely related taxa [30,31,32]. Double-digest restriction site-associated DNA sequencing (ddRAD-seq), which combines enzymatic fragmentation of genomic DNA with high-throughput sequencing, is a commonly used technique to generate a large number of genome-wide SNPs in non-model taxa [33]. This approach has proven effective for delimiting species boundaries among closely related species [21,34,35].

**Figure 1:**
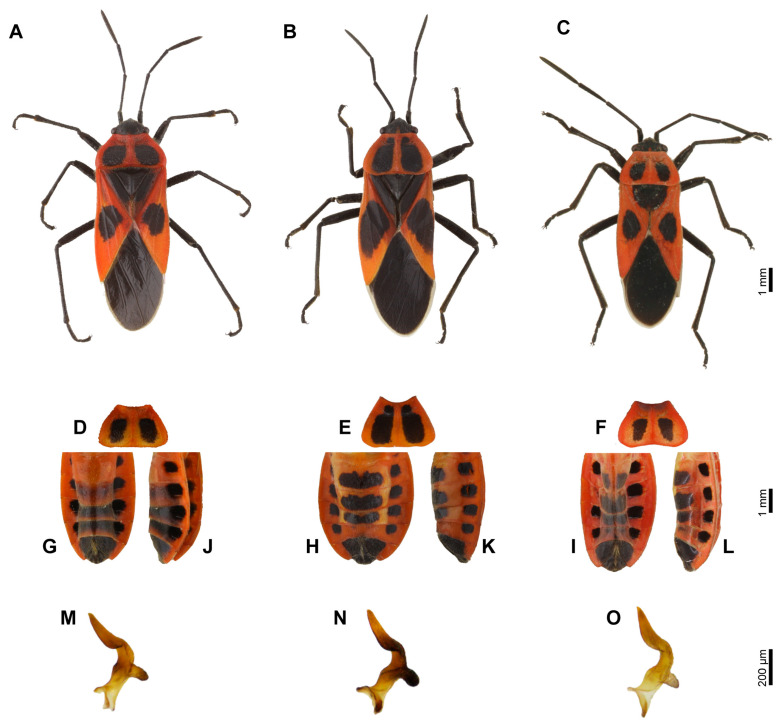
Morphological comparison between *T. sinensis*, *T. cruciger*, and the “intermediate form”. (**A**–**C**) Dorsal views of *T. sinensis* (female), *T. cruciger* (female), and the “intermediate form” (male); (**D**–**F**) dorsal views of the pronotum of *T. sinensis* (female), *T. cruciger* (female), and the “intermediate form” (female); (**G**–**I**) ventral views of the partial abdominal segments of *T. sinensis* (female), *T. cruciger* (female), and the “intermediate form” (female); (**J**–**L**) lateral views of the partial abdominal segments of *T. sinensis* (female), *T. cruciger* (female), and the “intermediate form” (female); (**M**–**O**) dorsal view of left parameres of *T. sinensis*, *T. cruciger*, and the “intermediate form”.

The genus *Tropidothorax* Bergroth, 1894 is mainly distributed in the Oriental region, with a total of 11 recorded species [36]. Among them, *Tropidothorax cruciger* (Motschulsky, 1860) and *Tropidothorax sinensis* (Reuter, 1888) are similar in morphology [37,38]. They can be distinguished based on the dark pattern on the pronotum and coriaceous part of hemelytra and the separation between the central black stripe and the lateral black spots on the abdomen [39,40]. However, the identification of these two species is often complicated by the presence of the “intermediate form”, which exhibits ventral characteristics similar to *T. cruciger* and dorsal characteristics resembling *T. sinensis* (Figure 1). Based on morphological features alone, individuals of this “intermediate form” cannot be reliably assigned to either *T. cruciger* or *T. sinensis*. To date, taxonomic studies of *T. sinensis* and the “intermediate form” have relied exclusively on morphological data, with no integration of molecular evidence. As a result, the taxonomic status of the “intermediate form” and the species boundaries between *T. cruciger* and *T. sinensis* remain unresolved.

In this study, morphological characteristics were used as an initial guide for species identification. We subsequently conducted species delimitation analyses based on both the COI fragment and genome-wide SNPs, in addition to niche comparison. The objectives of this study are (1) to clarify the taxonomic status of the “intermediate form” between *T. cruciger* and *T. sinensis* and (2) to delineate precise species boundaries between *T. cruciger* and *T. sinensis*.

## 2. Materials and Methods

### 2.1. Sampling and DNA Extraction

**Figure 2:**
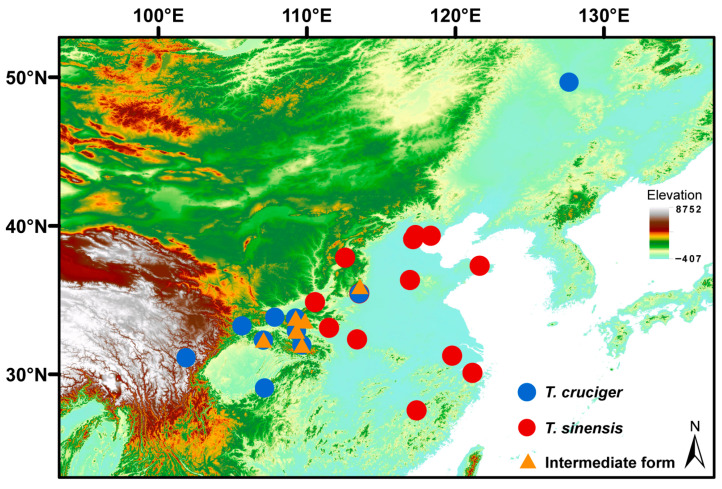
Geographical map of the sampling sites. The blue dots represent *T. cruciger*, the red dots represent *T. sinensis*, and the orange dots represent the “intermediate form”.

A total of 96 individuals belonging to *T. cruciger* (35), *T. sinensis* (41) and the “intermediate form” (20) were collected and analysed in this study (Figure 2, Table S1). Specimens were identified using morphological taxonomic keys [39,40]. All specimens were preserved in 100% ethanol and stored at −20 °C at the Institute of Entomology at Nankai University (Tianjin, China). Total genomic DNA was extracted from the thoracic muscle using a Universal Genomic DNA Kit (CWBIO, Beijing, China). Detailed information of samples and localities is provided in Table S1.

### 2.2. Acquisition of Molecular Datasets

All samples were sequenced for the COI fragment using the universal primers LCO1490 and HCO2198 [26]. The PCR procedure consisted of an initial denaturation step at 94 °C for 2 min, followed by 33 cycles of 30 s at 94 °C, 30 s at 50 °C, and 45 s at 72 °C. Finally, a final extension was performed at 72 °C for 8 min. PCR products were sequenced using Sanger sequencing at the Beijing Genomics Institute (BGI). Sequences were aligned using the MAFFT algorithm on the EMBL-EBI website (https://www.ebi.ac.uk/jdispatcher/msa/mafft, accessed on 1 November 2023) [41]. Finally, we generated a COI dataset consisting of 645 nucleotide sites obtained from 96 individuals.

A total of 78 samples were selected for ddRAD-seq. The ddRAD libraries were prepared using the protocol described by Peterson et al. (2012) [33] with minor adjustments detailed below. A total of 600 ng of DNA from each sample was double-digested with EcoRI and MspI restriction enzymes. Ligation products were pooled and then purified with AMPure XP Beads. Purified products were amplified with 8–10 cycles using PCR with Illumina indexed primers. Finally, the amplified products were purified and size-selected for fragments between 250 and 600 bp using the Pippin Prep system. Each library was quantified using an Agilent 2100 Bioanalyzer (Agilent, San Jose, California, United States) and sequenced using the Illumina NovaSeq X Plus platform.

Raw reads were demultiplexed and processed using the ipyrad v.0.9.42 pipeline [42]. We used the de novo assembly method with a pair-ddRAD datatype. Bases with Phred scores below 33 were replaced by Ns, and any reads with more than 5 Ns were removed. A clustering threshold of 0.85 was used to identify homologous reads. Five bases were trimmed from the beginning and end of R1 and R2 reads to unify the length of reads. Five bases were also trimmed from the edges of the final aligned loci. The remaining parameters were set to default values. Finally, the dataset was filtered to retain only loci present in at least 90% of individuals, generating a ddRAD SNP dataset with 78 individuals and 52,348 SNPs. To avoid linkage across sites within the same locus, one random SNP was sampled from each locus, and a ddRAD USNP dataset with 78 individuals and 2064 unlinked SNPs was generated.

### 2.3. Species Delimitation Analyses Based on COI Fragment

We employed four species delimitation methods based on the COI dataset: Automatic Barcode Gap Discovery (ABGD) [43], Assemble Species by Automatic Partitioning (ASAP) [44], Bayesian Poisson Tree Processes (bPTP) [45] and Generalized Mixed Yule Coalescent model (GMYC) [46,47]. ABGD analysis was conducted on the web server (https://bioinfo.mnhn.fr/abi/public/abgd/abgdweb.html, accessed on 6 November 2025) using the Kimura 2-P (K80) distance model and others keep default. ASAP analysis was performed on the web server (https://bioinfo.mnhn.fr/abi/public/asap, accessed on 6 November 2025) with the Kimura 2-P (K80) distance model. A non-ultrametric tree as the input file for the bPTP analysis was reconstructed using IQ-TREE 2.2.0 [48] without an outgroup. Then, bPTP analysis was conducted on the species delimitation web server for bPTP (https://species.h-its.org/ptp/, accessed on 6 November 2025). An ultrametric tree as the input file for GMYC analysis was reconstructed using BEAST 2.6.6 [49] without an outgroup under a Yule model prior and a strict clock model. The Markov chain Monte Carlo (MCMC) chains were run for 1 million generations, sampling every 10,000 generations. Tracer 1.7 [50] was used to assess the convergence of runs. Consensus trees were generated by TreeAnnotator 2.6.6 [49], with a burn-in percentage at 10%. Then, GMYC analysis was performed on the web server (http://species.h-its.org/gmyc/, accessed on 6 November 2025) under a single-threshold method. In addition, intra- and interspecific genetic distances based on the COI dataset were calculated using MEGA X [51] under the Kimura-2-parameter (K2P) nucleotide substitution model.

### 2.4. Species Delimitation Analyses Based on Genome-Wide SNPs Data

The Bayes Factor Delimitation (BFD*) method [52] implemented in SNAPP [53] and BEAST v2.6.6 [49], based on the ddRAD USNP dataset, was used to compare possible species delimitation hypotheses. A total of three possible species delimitation models were compared based on the marginal likelihood estimate (MLE), and the detailed description for each model is provided in Table S2. The MLE for each model was obtained using a path-sampling analysis with 50 steps, a chain length of 1,000,000 generations and a 10% burn-in. The calculation of Bayes factors (BFs) and ranking of different models followed the guidelines outlined in the BFD* tutorial.

In addition, we employed three methods to infer genetic clustering and admixture. Phylogenetic networks were constructed based on the ddRAD SNP dataset using the neighbour-net method based on pairwise uncorrected *p* distances among samples, which was implemented in SplitsTree 4.14.5 [54]. Principal component analysis (PCA) was performed using the R package adegenet 2.1.10 [55] based on the ddRAD USNP dataset. Bayesian clustering was performed in STRUCTURE 2.3.4 [56] based on the ddRAD USNP dataset. The program was run under an admixture model with a burn-in of 100,000 generations and a run length of 500,000 generations. K values from 1 to 10 were examined, with ten replicate runs for each K value. The best K value was evaluated using STRUCTURE HARVESTER 0.6.94 [57] with the Delta K method. CLUMPP 1.1.2 [58] was used to summarize runs for each K value. A plot was constructed with DISTRUCT 1.1 [59].

### 2.5. Principal Component Analysis of Environmental Variables

We compared the niches among *T. cruciger*, *T. sinensis* and the “intermediate form” based on 19 climatic variables and elevation. The 19 climatic variables and elevation were extracted from the WorldClim database (http://www.worldclim.org/, accessed on 13 May 2024) using the R package raster 3.6.32 [60]. Then, the extracted climatic variables and elevation were analysed by principal component analysis using the R package FactoMineR 2.12 [61].

## 3. Results

### 3.1. Datasets

For the COI dataset, a total of 82 polymorphic sites were detected from 96 individuals, including 72 parsimony informative sites and 10 singleton variable sites. For the ddRAD dataset, a total of 492,964,084 raw reads were generated from 78 individuals. After low-quality reads were filtered out, 490,868,842 reads were retained, ranging from 998,191 to 20,246,157 and averaging 6,293,190 reads per sample. After de novo assembly and SNP calling, our two final datasets (dataset file names: ddRAD SNP dataset and ddRAD USNP dataset) consisted of 2064 loci, 52,348 SNPs and 2064 unlinked SNPs, with 6.86% missing sites.

### 3.2. Species Delimitation Results Based on COI Fragment

**Figure 3:**
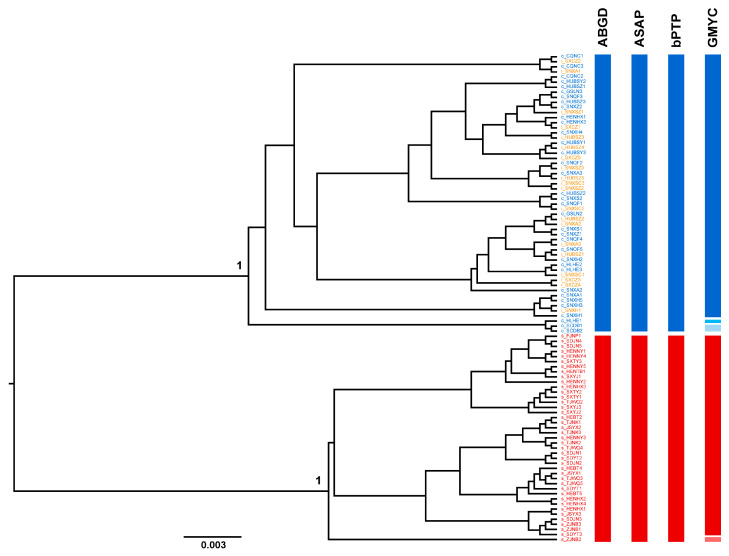
Summary of species delimitation results based on the COI fragment. The ultrametric tree on the left was reconstructed using BEAST. The columns on the right are the results of four molecular delimitation methods (ABGD, ASAP, bPTP and GMYC). The putative species inferred by the molecular delimitation method are shown in different colors.

Based on the COI dataset, most species delimitation methods consistently identified two distinct groups (Figure 3). One group comprised all individuals of *T. cruciger* and the “intermediate form”, while the other included all individuals of *T. sinensis*. This grouping pattern was strongly supported by the phylogenetic tree, which recovered each group as a highly supported monophyletic clade. Both distance-based methods (ABGD and ASAP) yielded two molecular operational taxonomic units (MOTUs), corresponding exactly to these two groups (Tables S3 and S4). The bPTP analysis further supported this delimitation scenario (Table S5). In contrast, the GMYC analysis retrieved five MOTUs, and details regarding the individuals in each MOTU are provided in Figure 3 and Figure S1.

The intraspecific genetic distances of *T. cruciger*, *T. sinensis*, “intermediate form” and *T. cruciger* + “intermediate form” were 0–2.38%, 0–2.06%, 0–0.78% and 0–2.38%, respectively. The interspecific genetic distance between *T. cruciger* and the “intermediate form” was 0.28%, which is significantly smaller than the genetic distances between *T. cruciger* and *T. sinensis* (9.26%) and between *T. sinensis* and the “intermediate form” (9.17%) (Table 1).

### 3.3. Species Delimitation Results Based on Genome-Wide SNPs Data

The BFD* analysis of the ddRAD USNP dataset showed that Model A (Two species: *T. cruciger* and the “intermediate form” comprise a single species; *T. sinensis* represents a separate species) obtained the highest MLE (Table S2). The BF values were larger than 10 (Table S2), indicating that Model A was strongly supported.

**Figure 4:**
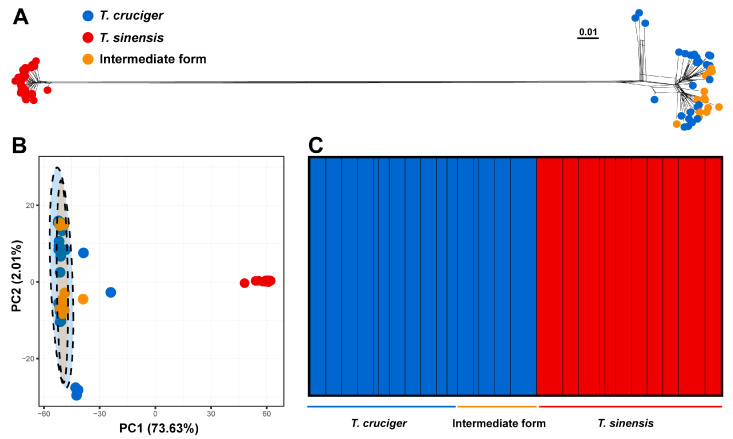
Genetic clustering results based on genome-wide SNPs. (**A**) Phylogenetic networks based on the ddRAD SNP dataset. (**B**) PCA based on the ddRAD USNP dataset. (**C**) STRUCTURE analysis based on the ddRAD USNP dataset (the best value K = 2).

The phylogenetic networks based on the ddRAD SNP dataset and the PCA based on the ddRAD USNP dataset consistently revealed two distinct genetic clusters. One cluster comprised *T. cruciger* and the “intermediate form”, while the other consisted of *T. sinensis* (Figure 4). In the PCA, the first and second principal components represented 73.63% and 2.01% of the genetic variation, respectively (Figure 4). The optimal K value in the STRUCTURE analysis was identified as 2 (Figure S2). When K = 2, individual assignments to clusters were consistent with the groupings observed in the phylogenetic networks and PCA. No genetic admixture was detected between *T. cruciger* + “intermediate form” and *T. sinensis* (Figure 4).

### 3.4. Principal Component Analysis of Environmental Variables

**Figure 5:**
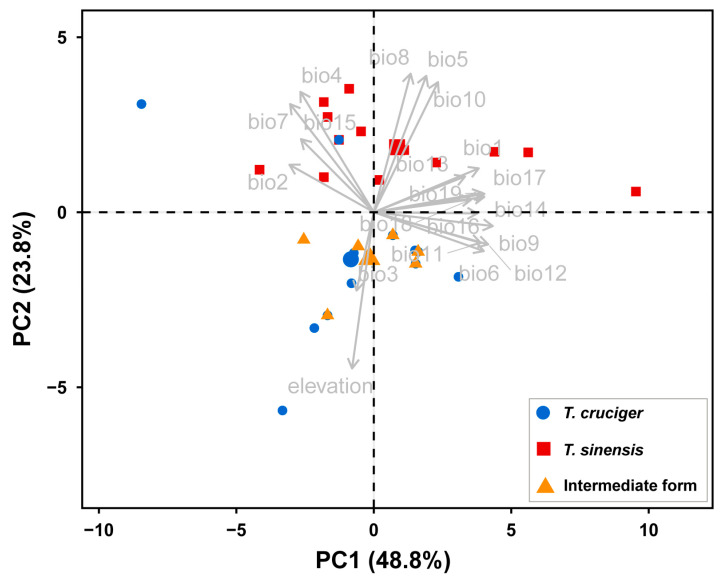
Principal component analysis (PCA) of environmental factors associated with *T. sinensis*, *T. cruciger*, and the “intermediate form”.

In the PCA of 19 climatic variables and elevation, the first two components explained 72.6% of the overall variance. According to the variable contribution values, PC1 was mainly associated with mean temperature of the driest quarter (BIO9), annual precipitation (BIO12), and precipitation of the driest quarter (BIO17), whereas PC2 was mainly associated with elevation, mean temperature of the wettest quarter (BIO8), and maximum temperature of the warmest month (BIO5). The PCA results showed that *T. cruciger* and the “intermediate form” clustered together with respect to PC1 and PC2, indicating their similar environmental space. In contrast, *T. sinensis* can be separated from both *T. cruciger* and the “intermediate form”, with the exception of one site in *T. cruciger* (Figure 5).

## 4. Discussion

In this study, we initially identified species based on morphological taxonomic keys. Although *T. cruciger* and *T. sinensis* are morphologically similar, they can be distinguished by specific traits such as the dark pattern on the pronotum and coriaceous part of hemelytra, as well as the separation between the median black band and the lateral black spots on the abdomen [40]. As expected, these morphological traits allow for clear and straightforward discrimination between the two species. However, we encountered a group of specimens representing an “intermediate form” that exhibits morphological similarities to one or the other species to varying degrees. In these intermediate specimens, the black spots on pronotum and coriaceous part of hemelytra are small to medium in size—characteristic of *T. sinensis*—whereas the separation between the central black stripe and lateral black spots on the abdomen aligns with traits typical of *T. cruciger* (Figure 1). Based solely on morphological features, it is difficult to assign these intermediate forms definitively to either *T. cruciger* or *T. sinensis*.

To address the above issue, we conducted species delimitation analyses using both the COI fragment and genome-wide SNPs. Most distance-based and tree-based species delimitation methods (ABGD, ASAP, bPTP) using the COI fragment suggested that *T. cruciger* and the “intermediate form” comprise a single species, with *T. sinensis* representing a distinct species. The GMYC analysis based on the COI fragment retrieved five MOTUs, resulting in the over-splitting of both *T. cruciger* and *T. sinensis*. This is consistent with the known tendency of the GMYC method to over-split species, which often arises from low genetic divergence between lineages, overlapping intra- and interspecific genetic distances, or the absence of reciprocal monophyly [62,63]. The interspecific genetic distance between *T. cruciger* and the “intermediate form” is only 0.28%, which is significantly smaller than that between *T. cruciger* and *T. sinensis* (9.26%) and between *T. sinensis* and the “intermediate form” (9.17%). Furthermore, the maximum intraspecific distances observed in *T. cruciger* (2.38%) and *T. sinensis* (2.06%) both exceed the interspecific distance between *T. cruciger* and the “intermediate form” (0.28%). Notably, when individuals of *T. cruciger* and the “intermediate form” are considered as a single species, a distinct barcode gap emerges between interspecific and intraspecific genetic distances. The taxonomic status of the “intermediate form” was also confirmed using genome-wide SNP data. BFD* analysis based on the ddRAD USNP dataset strongly supported the two-species model, with the combination of *T. cruciger* and the “intermediate form” as one species and *T. sinensis* as the other, yielding the highest MLE. Population genetic clustering analyses using phylogenetic networks, PCA and STRUCTURE also revealed two distinct genetic clusters. One cluster comprised *T. cruciger* and the “intermediate form”, while the other consisted exclusively of *T. sinensis*. In addition, environmental-related analyses also showed that *T. cruciger* and the “intermediate form” clustered together, consistent with their highly similar ecological requirements and supporting their classification as a single species.

Based on the results of species delimitation obtained in this study, we clarify the taxonomic status of the “intermediate form” between *T. cruciger* and *T. sinensis*. It is concluded that this “intermediate form” should be recognized as a morphological variant of *T. cruciger*.

## 5. Conclusions

In this study, we conducted an integrative taxonomic analysis of two species, *T. cruciger* and *T. sinensis*, by combining morphological traits, molecular-based species delimitation and ecological niche comparison. Species identification has been complicated by the presence of the “intermediate form”, which is difficult to assign unequivocally to either *T. cruciger* or *T. sinensis* based on morphology alone. Results from species delimitation using both the COI fragment and genome-wide SNPs, together with environmental-related analysis, strongly supported that *T. cruciger* and the “intermediate form” represent a single species, while *T. sinensis* constitutes a distinct species. Under this species delimitation scenario, a clear barcode gap was observed between interspecific and intraspecific genetic distances.

## Figures and Tables

**Table 1:** Intraspecific and interspecific genetic distances based on the COI dataset. Abbreviations: TC, *T. cruciger*; TS, *T. sinensis*; TI, the “intermediate form”.

Gene	Intraspecific Distance (%)				Interspecific Distance (%)		
	TC	TS	TI	TC + TI	TC − TS	TC − TI	TS − TI
COI	0–2.38%	0–2.06%	0–0.78%	0–2.38%	9.26%	0.28%	9.17%

## Data Availability

Individual RAD sequences files are available in GenBank (accession numbers: SAMN53285687–SAMN53285764). COI sequences generated in the present study are available in GenBank (accession numbers: PX518918–PX519013).

## Supplementary Materials

The following supporting information can be downloaded at https://www.mdpi.com/article/10.3390/insects17010037/s1. Table S1. Sample information of *T. cruciger*, *T. sinensis* and the “intermediate form” in the present study. Table S2. Comparison of different species delimitation models. Table S3. Species delimitation results using the ABGD method based on the COI dataset. Table S4. Species delimitation results using the ASAP method based on the COI dataset. Table S5. Species delimitation results using the bPTP method based on the COI dataset. Figure S1. Species delimitation results using the GMYC method based on the COI dataset. (A) Species defined by single threshold GMYC model. (B) The *X*-axis represents time, the *Y*-axis represents the number of branches, and the red vertical line represents the conversion time of the population and species. (C) The *X*-axis represents time, and the *Y*-axis represents the log-likelihood value of the single threshold GMYC model. Figure S2. Rate of change of likelihood (Delta K) across multiple runs of STRUCTURE.

## Abbreviations

The following abbreviations are used in this manuscript:

COI	Cytochrome c oxidase subunit I
BFD*	Bayes Factor Delimitation
ddRAD	Double-digest restriction site-associated DNA
