# Supplementary material for: Integrative Taxonomy Clarifies the Taxonomic Status of the Morphologically Intermediate Form Between Tropidothorax cruciger and T. sinensis (Hemiptera: Lygaeidae)
Source: Insects. 2025 Dec 26;17(1):37. doi: 10.3390/insects17010037 (PMC12841810; doi:10.3390/insects17010037)
Supplement: Supplementary file 1 [file insects-17-00037-s001.zip › insects-4025397-supplementary.pdf]

Supporting Information

Table S1. Sample information of *T. cruciger*, *T. sinensis* and the "intermediate form" in the present study.

Species	Locality	Population names	Sample size	Longitude	Latitude	Collection data
T. cruciger	Sunwu County, Heihe City, Heilongjiang Province	c_HLHE	3	127.65	49.67	2023/8/23
T. cruciger	Duchuan Village, Yerengu, Shiyan City, Hubei Province	c_HUBSY	3	110.72	31.89	2017/7/17
T. cruciger	Yangquanzi Village, Quanxi Town, Zhuxi County, Shiyan City, Hubei Province	c_HUBSZ	3	109.67	31.98	2017/7/12
T. cruciger	Bailiu Town, Xunyang County, Ankang City, Shaanxi Province	c_SNXA	3	109.30	32.92	2014/8/1
T. cruciger	Wuxiang, Hanzhong City, Shaanxi Province	c_SNXH	5	107.07	32.32	2014/7/8
T. cruciger	Yingpan Town, Zhashui County, Shangluo City, Shaanxi Province	c_SNXS	2	109.24	33.79	2014/7/30
T. cruciger	Damianpo, Nanchuan District, Chongqing Municipality	c_CQNC	3	107.15	29.08	2022/7/21
T. cruciger	Anmenkou Town, Kang County, Longnan City, Gansu Province	c_GSLN	2	105.63	33.27	2023/6/29
T. cruciger	Xigou Village, Huixian City, Henan Province	c_HENHX	2	113.53	35.45	2014/8/21
T. cruciger	Huaerping Village, Houzhenzi Town, Zhouzhi County, Shaanxi Province	c_SNXZ	2	107.83	33.85	2013/8/26
T. cruciger	Peier Village, Badi Town, Danba County, Sichuan Province	c_SCDB	2	101.87	31.13	2018/8/29
T. cruciger	Longtanyicun Village, Fenghuang Town, Zhashui County, Qinling, Shaanxi Province	c_SNQF	5	109.35	33.51	2014/6/26
T. sinensis	Zhengzhuangzi Village, Caofeidian District, Tangshan City, Hebei Province	s_HEBT	3	118.34	39.33	2020/6/6

T. sinensis	Xichuan County, Nanyang City, Henan Province	s_HENNY	5	111.49	33.14	2018/11/29
T. sinensis	Xigou Village, Huixian City, Henan Province	s_HENHX	4	113.53	35.45	2014/8/21
T. sinensis	Disanyu Village, Wande Town, Changqing District, Jinan City, Shandong Province	s_SDJN	5	116.95	36.35	2019/7/13
T. sinensis	Rongshengzhuang Village, Yongji City, Shanxi Province	s_SXYJ	3	110.55	34.86	2018/8/5
T. sinensis	Kunyu Mountain, Yantai City, Shandong Province	s_SDYT	3	121.63	37.31	2018/8/4
T. sinensis	Nankai University, Tianjin City	s_TJNK	3	117.17	39.11	2018/10/1
T. sinensis	Bingzhou East Street, Yingze District, Taiyuan City, Shanxi Province	s_SXTY	3	112.57	37.85	2018/6/1
T. sinensis	Aoli, Yuyao North Station, Ningbo City, Zhejiang Province	s_ZJNB	3	121.16	30.10	2018/7/29
T. sinensis	Zhangyang Ganhoushan Group, Dingshu Town, Yixing City, Jiangsu Province	s_JSYX	3	119.78	31.26	2019/7/20
T. sinensis	Raoping Village, Nanping, Huixian, Fujian Province	s_FJNP	1	117.40	27.58	2018/7/21
T. sinensis	Buddhist College, Tongbai County, Henan Province	s_HENTB	1	113.39	32.36	2019/8/3
T. sinensis	Xi'anzi Village, Wuqing District, Tianjin Municipality	s_TJWQ	4	117.31	39.38	2019/6/21
“intermediate form”	Yangquanzi Village, Quanxi Town, Zhuxi County, Shiyan City, Hubei Province	i_HUBSZ	5	109.67	31.98	2017/7/12
“intermediate form”	Hongdou Gorge, Taihangshan Grand Canyon, Changzhi City, Shanxi Province	i_SXCZ	5	113.57	35.92	2018/8/9
“intermediate form”	Bailiu Town, Xunyang County, Ankang City, Shaanxi Province	i_SNXA	3	109.30	32.92	2014/8/1
“intermediate form”	Wuxiang, Hanzhong City, Shaanxi Province	i_SNXH	1	107.07	32.32	2014/7/8
“intermediate form”	Chengguan Town, Shanyang County, Shaanxi Province	i_SNXSC	3	109.87	33.61	2014/8/9
“intermediate form”	Yingpan Town, Zhashui County, Shangluo City, Shaanxi Province	i_SNXSZ	3	109.24	33.79	2014/7/30

Table S2. Comparison of different species delimitation models.

Model	Species	MLE	Rank	BF
A	Two species: *T. cruciger* + the "intermediate form", *T. sinensis*	-6236.42	1	–
C	Three species: *T. cruciger*, *T. sinensis*, the "intermediate form"	-7525.92	2	2579
B	Two species: *T. sinensis* + the "intermediate form", *T. cruciger*	-13595.31	3	12138.78

MLE: Marginal likelihood estimate

BF: Bayes factor

Table S3. Species delimitation results using the ABGD method based on the COI dataset.

**Croup**	**Sample number**	**Sample names**
Group[1]	55	c_CQNC1, c_CQNC2, c_CQNC3, c_GSLN2, c_GSLN3, c_HENHX1, c_HENHX3, c_HLHE1, c_HLHE2, c_HLHE3, c_HUBSY1, c_HUBSY2, c_HUBSY3, c_HUBSZ1, c_HUBSZ2, c_HUBSZ3, c_SCDB1, c_SCDB2, c_SNQF1, c_SNQF2, c_SNQF3, c_SNQF4, c_SNQF5 ,c_SNXA1, c_SNXA2 , c_SNXA3, c_SNXH1, c_SNXH2 , c_SNXH3, c_SNXH4, c_SNXH5, c_SNXS1, c_SNXS2, c_SNXZ1, c_SNXZ2, i_HUBSZ1, i_HUBSZ2, i_HUBSZ3, i_HUBSZ4, i_HUBSZ5, i_SNXA1, i_SNXA2, i_SNXA3, i_SNXH1, i_SNXSC1, i_SNXSC2, i_SNXSC3, i_SNXSZ1, i_SNXSZ2, i_SNXSZ3, i_SXCZ1, i_SXCZ2, i_SXCZ3, i_SXCZ4, i_SXCZ5
Group[2]	41	s_FJNP1, s_HEBT2, s_HEBT4, s_HEBT5, s_HENHX1, s_HENHX2, s_HENHX3, s_HENHX4, s_HENNY1, s_HENNY2, s_HENNY3, s_HENNY4, s_HENNY5, s_HENTB1, s_JSYX1, s_JSYX2, s_JSYX3, s_SDJN1, s_SDJN2, s_SDJN3, s_SDJN4, s_SDJN5, s_SDYT1, s_SDYT2, s_SDYT3, s_SXTY1, s_SXTY2, s_SXTY3, s_SXYJ1, s_SXYJ2, s_SXYJ3, s_TJNK1, s_TJNK2, s_TJNK3, s_TJWQ2, s_TJWQ3, s_TJWQ4, s_TJWQ5, s_ZJNB1, s_ZJNB2, s_ZJNB3

Table S4. Species delimitation results using the ASAP method based on the COI dataset.

Subset	Sample number	Sample names
Subset[1]	55	c_CQNC1, c_CQNC2, c_CQNC3, c_GSLN2, c_GSLN3, c_HENHX1, c_HENHX3, c_HLHE1, c_HLHE2, c_HLHE3, c_HUBSY1, c_HUBSY2, c_HUBSY3, c_HUBSZ1, c_HUBSZ2, c_HUBSZ3, c_SCDB1, c_SCDB2, c_SNQF1, c_SNQF2, c_SNQF3, c_SNQF4, c_SNQF5 ,c_SNXA1, c_SNXA2 , c_SNXA3, c_SNXH1, c_SNXH2 , c_SNXH3, c_SNXH4, c_SNXH5, c_SNXS1, c_SNXS2, c_SNXZ1, c_SNXZ2, i_HUBSZ1, i_HUBSZ2, i_HUBSZ3, i_HUBSZ4, i_HUBSZ5, i_SNXA1, i_SNXA2, i_SNXA3, i_SNXH1, i_SNXSC1, i_SNXSC2, i_SNXSC3, i_SNXSZ1, i_SNXSZ2, i_SNXSZ3, i_SXCZ1, i_SXCZ2, i_SXCZ3, i_SXCZ4, i_SXCZ5
Subset[2]	41	s_FJNP1, s_HEBT2, s_HEBT4, s_HEBT5, s_HENHX1, s_HENHX2, s_HENHX3, s_HENHX4, s_HENNY1, s_HENNY2, s_HENNY3, s_HENNY4, s_HENNY5, s_HENTB1, s_JSYX1, s_JSYX2, s_JSYX3, s_SDJN1, s_SDJN2, s_SDJN3, s_SDJN4, s_SDJN5, s_SDYT1, s_SDYT2, s_SDYT3, s_SXTY1, s_SXTY2, s_SXTY3, s_SXYJ1, s_SXYJ2, s_SXYJ3, s_TJNK1, s_TJNK2, s_TJNK3, s_TJWQ2, s_TJWQ3, s_TJWQ4, s_TJWQ5, s_ZJNB1, s_ZJNB2, s_ZJNB3

Table S5. Species delimitation results using the bPTP method based on the COI dataset.

Species	Support	Sample number	Sample names
Species 1	0.996	41	s_FJNP1, s_HEBT2, s_HEBT4, s_HEBT5, s_HENHX1, s_HENHX2, s_HENHX3, s_HENHX4, s_HENNY1, s_HENNY2, s_HENNY3, s_HENNY4, s_HENNY5, s_HENTB1, s_JSYX1, s_JSYX2, s_JSYX3, s_SDJN1, s_SDJN2, s_SDJN3, s_SDJN4, s_SDJN5, s_SDYT1, s_SDYT2, s_SDYT3, s_SXTY1, s_SXTY2, s_SXTY3, s_SXYJ1, s_SXYJ2, s_SXYJ3, s_TJNK1, s_TJNK2, s_TJNK3, s_TJWQ2, s_TJWQ3, s_TJWQ4, s_TJWQ5, s_ZJNB1, s_ZJNB2, s_ZJNB3
Species 2	0.961	55	c_CQNC1, c_CQNC2, c_CQNC3, c_GSLN2, c_GSLN3, c_HENHX1, c_HENHX3, c_HLHE1, c_HLHE2, c_HLHE3, c_HUBSY1, c_HUBSY2, c_HUBSY3, c_HUBSZ1, c_HUBSZ2, c_HUBSZ3, c_SCDB1, c_SCDB2, c_SNQF1, c_SNQF2, c_SNQF3, c_SNQF4, c_SNQF5 ,c_SNXA1, c_SNXA2 , c_SNXA3, c_SNXH1, c_SNXH2 , c_SNXH3, c_SNXH4, c_SNXH5, c_SNXS1, c_SNXS2, c_SNXZ1, c_SNXZ2, i_HUBSZ1, i_HUBSZ2, i_HUBSZ3, i_HUBSZ4, i_HUBSZ5, i_SNXA1, i_SNXA2, i_SNXA3, i_SNXH1, i_SNXSC1, i_SNXSC2, i_SNXSC3, i_SNXSZ1, i_SNXSZ2, i_SNXSZ3, i_SXCZ1, i_SXCZ2, i_SXCZ3, i_SXCZ4, i_SXCZ5

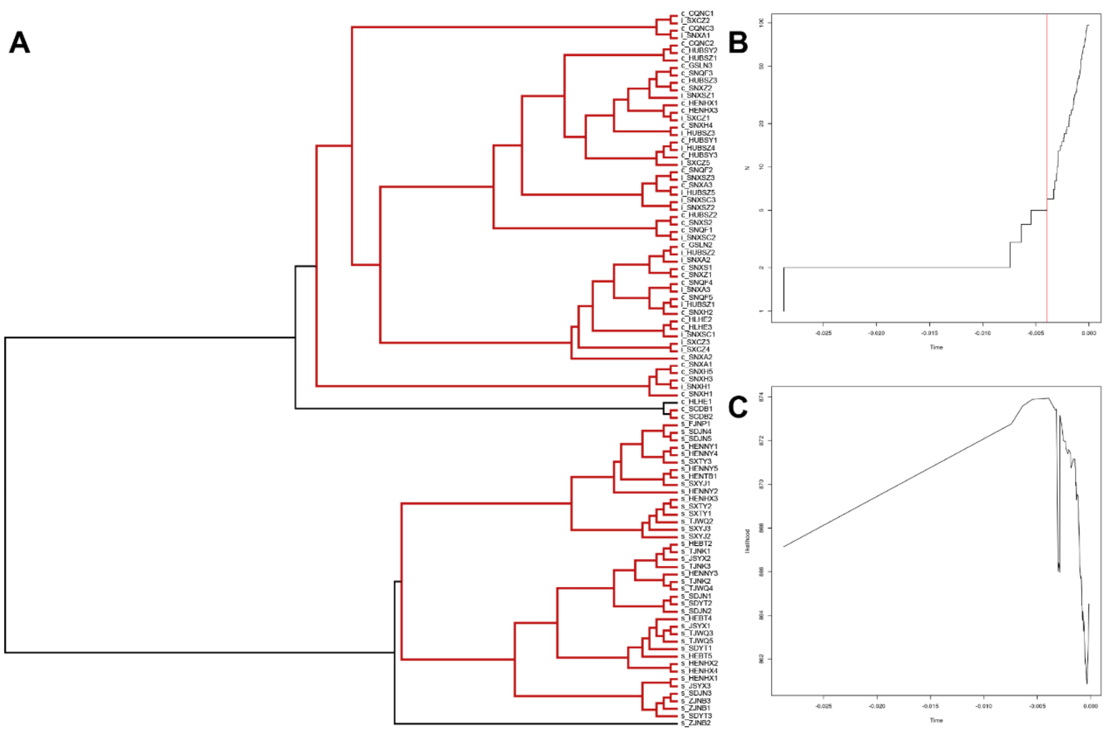

Figure S1. Species delimitation results using the GMYC method based on the COI dataset. (A) Species defined by single threshold GMYC model. (B) The X-axis represents time, the Y-axis represents the number of branches, and the red vertical line represents the conversion time of the population and species. (C) The X-axis represents time, and the Y-axis represents log-likelihood value of single threshold GMYC model.

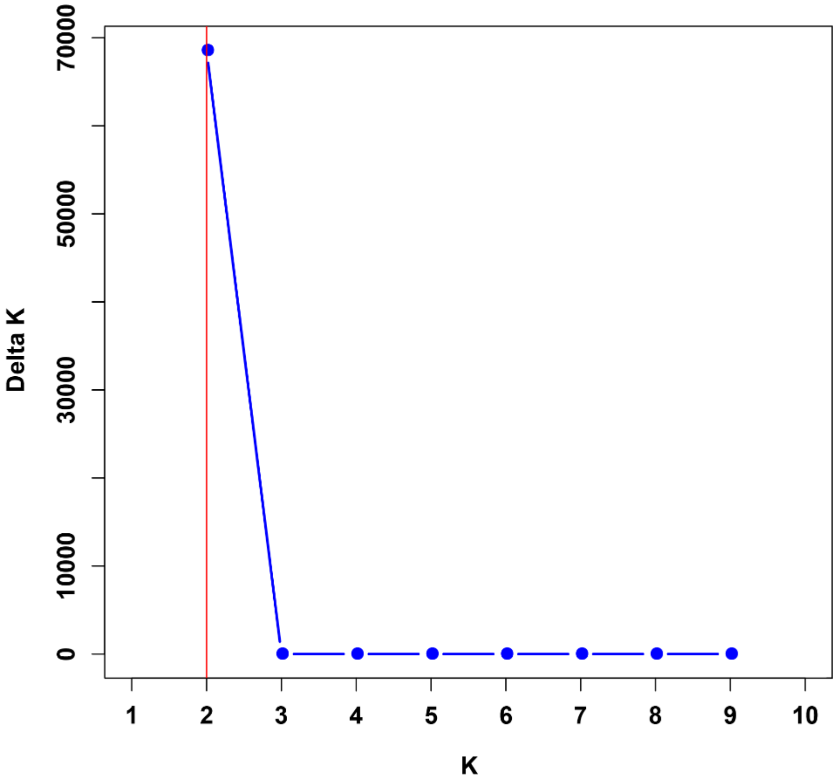

Figure S2. Rate of change of likelihood (Delta K) across multiple runs of STRUCTURE.
